# Changes in Muscle Stress and Sarcomere Adaptation in Mice Following Ischemic Stroke

**DOI:** 10.3389/fphys.2020.581846

**Published:** 2020-12-17

**Authors:** Liang-Ching Tsai, Yi-Ning Wu, Shu Q. Liu, Li-Qun Zhang

**Affiliations:** ^1^Department of Physical Therapy, Georgia State University, Atlanta, GA, United States; ^2^Department of Physical Therapy and Kinesiology, University of Massachusetts Lowell, Lowell, MA, United States; ^3^Department of Biomedical Engineering, Northwestern University, Evanston, IL, United States; ^4^Department of Physical Therapy and Rehabilitation Science, University of Maryland, Baltimore, MD, United States; ^5^Department of Orthopaedics, University of Maryland, Baltimore, MD, United States; ^6^Department of Bioengineering, University of Maryland, College Park, MD, United States

**Keywords:** force microscope, soleus, muscle, in-vivo muscle tension, mouse stroke, sarcomerogenesis

## Abstract

While abnormal muscle tone has been observed in people with stroke, how these changes in muscle tension affect sarcomere morphology remains unclear. The purpose of this study was to examine time-course changes in passive muscle fiber tension and sarcomeric adaptation to these changes post-ischemic stroke in a mouse model by using a novel *in-vivo* force microscope. Twenty-one mice were evenly divided into three groups based on the time point of testing: 3 days (D3), 10 days (D10), and 20 days (D20) following right middle cerebral artery ligation. At each testing time, the muscle length, width, and estimated volume of the isolated soleus muscle were recorded, subsequently followed by *in-vivo* muscle tension and sarcomere length measurement. The mass of the soleus muscle was measured at the end of testing to calculate muscle density. Two-way ANOVA with repeated measures was used to examine the differences in each of the dependent variable among the three time-point groups and between the two legs. The passive muscle stress of the impaired limbs in the D3 group (27.65 ± 8.37 kPa) was significantly lower than the less involved limbs (42.03 ± 18.61 kPa; *p* = 0.05) and the impaired limbs of the D10 (48.92 ± 14.73; *p* = 0.03) and D20 (53.28 ± 20.54 kPa; *p* = 0.01) groups. The soleus muscle density of the impaired limbs in the D3 group (0.69 ± 0.12 g/cm^3^) was significantly lower than the less involved limbs (0.80 ± 0.09 g/cm^3^; *p* = 0.04) and the impaired limbs of the D10 (0.87 ± 0.12 g/cm^3^; *p* = 0.02) and D20 (1.00 ± 0.14 g/cm^3^; *p* < 0.01) groups. The D3 group had a shorter sarcomere length (2.55 ± 0.26 μm) than the D10 (2.83 ± 0.20 μm; *p* = 0.03) and D20 group (2.81 ± 0.15 μm; *p* = 0.04). These results suggest that, while ischemic stroke may cause considerable changes in muscle tension and stress, sarcomere additions under increased mechanical loadings may be absent or disrupted post-stroke, which may contribute to muscle spasticity and/or joint contracture commonly observed in patients following stroke.

## Introduction

Alteration in muscle properties due to motor dysfunction negatively impacts stroke survivors’ mobility and self-care and thus has been a major clinical concern when treating stroke patients. Patients post-stroke often show substantial changes in muscle tone/tension, including the initial flaccid phase (i.e., reduced muscle tension) starting shortly after a stroke and the subsequent development of muscle spasticity that increases muscle tension in the affected limbs (Dietz and Berger, 1983; Hufschmidt and Mauritz, 1985; Lee et al., 1987; Feit et al., 1989; Sinkjaer et al., 1993, 1996; Sinkjaer and Magnussen, 1994; Booth et al., 2001). Although altered muscle tension following stroke, such as spasticity, has been associated with changes in the muscle fiber and sarcomere properties (Lieber and Friden, 2002), the role of the altered muscle tension following stroke in sarcomeric adaptation remains unclear. Changes of passive muscle properties after stroke have been studied at the joint level (Chung et al., 2004, 2008), and ultrasound studies have demonstrated the muscle contractility and tendon characteristics post-stroke (Gao and Zhang, 2008; Zhao et al., 2008). Aforementioned research investigated muscle functions from a system level at a particular time (i.e., a cross-sectional study design); a better understanding through *in vivo* investigations of the changes in sarcomere morphology and muscle tension at several consequential time points may help advance clinical guidance (Smeulders and Kreulen, 2006).

Muscle cell adaptation (sarcomerogenesis) has been thought to be a process responsible for the maintenance of the optimal length and force-length relationship by adjusting the number of sarcomeres in series, so that the muscle could be used efficiently (Herring et al., 1984). The process of muscle cell adaptation is activated in response to the demand of workload as observed in previous studies involving immobilization and lengthening (Goldspink et al., 1974; Williams and Goldspink, 1978; Williams et al., 1999; Gajdosik, 2001; Caiozzo et al., 2002; Lindsey et al., 2002; Coutinho et al., 2004), hind-limb unloading (Kasper and Xun, 2000; Wang et al., 2006), and tenotomy (Baker and Hall-Craggs, 1978). However, little is known regarding changes in the intrinsic properties of muscle cells following stroke.

It has been suggested that an intact innervation, both descending and ascending pathways, is critical for proper adjustment/adaptation of sarcomere length, and sarcomerogenesis might occur at a slower rate if innervation is compromised (McLachlan and Chua, 1983; Huijing and Jaspers, 2005; Swaroop et al., 2009). For example, in a rat leg lengthening study with the groups of lengthening only, lengthening with Botox, lengthening with neurectomy, and sham surgery, the number of serial sarcomeres in the soleus muscle fibers of the lengthening only group had a significantly more addition of sarcomeres in series when compared to the other three groups (Swaroop et al., 2009). In children with cerebral palsy (CP), impaired sarcomeric adaptation to the stretching of the muscle during growth/development has been thought to be the cause of the observed increased sarcomere length in this neurological patient population (Dayanidhi and Lieber, 2014; Matthiasdottir et al., 2014; Mathewson and Lieber, 2015; Kinney et al., 2017). In ischemic stroke, however, it remains poorly understood whether and how the muscle cells can adapt in response to changes in muscle mechanical environment throughout pathology progression.

Quantitative investigations to evaluate muscle fiber tension *in vivo* and its influence on sarcomeric adaptation post-stroke are limited because of the difficulties in human subject testing. Pre-clinical rodent models allow researchers to study neurological disease and motor system dysfunction (Farr and Whishaw, 2002; Farr et al., 2006; Granziera et al., 2007). Mice with induced stroke demonstrated similar functional and motor deficits to what have been observed in stroke patients, such as locomotor activity, gait, and limb tone (Hunter et al., 2000; Li et al., 2004). Thus, the objective of this study was to investigate the sarcomeric adaptation and the changes in muscle tension following stroke using a mouse ischemic stroke model. We hypothesized that changes in muscle tension due to a neural injury would not lead to sarcomeric adaptation due to impaired innervation in mice following ischemic stroke.

## Materials and Methods

The animal use in this study was approved by the Institutional Animal Care and Use Committee at Northwestern University. All animals were handled based on the guidelines by the National Institutes of Health. All animals were provided with food and water *ad libitum* and were maintained on a 12-h light-dark cycle. Twenty-one C57L/6 male mice (The Jackson Laboratory, Bar Harbor, Maine, United States), weighing between 20 and 30 g, received a brain surgery to induce ischemic stroke in the right hemisphere by the right middle cerebral artery (MCA) occlusion. The MCA occlusion is a common and validated model to study ischemic stroke in mice and rats (Durukan and Tatlisumak, 2009) with functional deficits observed beyond 30 days following surgery (Liu et al., 2013). The detailed procedure is elucidated in the section below. Stroke mice were then randomly divided into three groups evenly (*N* = 7 per group) according to the time point of testing including 3 days (D3), 10 days (D10), and 20 days (D20) after the MCA occlusion.

### Brain Surgery to Induce Stoke in Mice

Before surgery to induce ischemic stroke, all mice were anesthetized through intraperitoneal injection of Xylazine (10 mg/kg of body weight) and Ketamine (100 mg/kg) and Cefazolin (11–12 mg/kg) was given subcutaneously. Under anesthesia, a 3-mm perpendicular incision was made to bisect a line between the right external auditory canal and the right eye. When the skull was exposed, a 2-mm hole was drilled at a location near the fusion of the zygomatic arch with the squamosal bone. The right MCA was identified and then occluded permanently using suture ligation while bilateral common carotid arteries were only ligated for 1 h (i.e., suture ligation removed after 1 h; Liu et al., 2013). Following surgery, mice were moved back to the animal housing facility with free water and food access. Mice with stroke were anesthetized for biomechanical testing of the soleus muscles either on the 3rd, 10th, or 20th day according to the group assignment following the brain surgery. After testing, the mice were euthanized and the isolated soleus muscles were removed for muscle mass measurement. Muscle density (mass divided by estimated volume; g/cm^3^) was then calculated. Estimated volume was derived from the width, length, and thickness of the isolated muscle by assuming the isolated muscle to be similar to a cylinder. Both the impaired (left) and the less involved (right) limb were tested in a random order.

### *In vivo* Quantification of Sarcomere Length and Sarcomere Number in the Soleus Muscle

**Figure 1:**
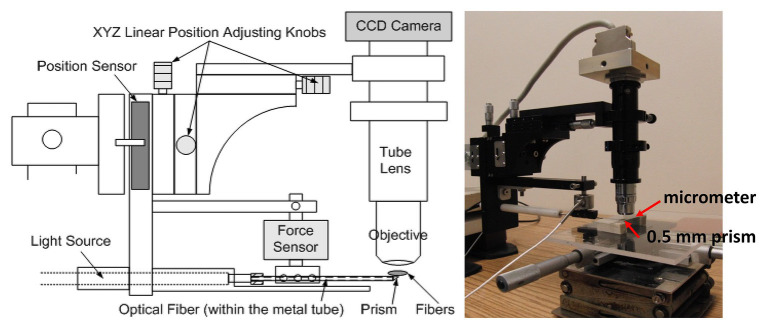
A schematic of the force microscope. Before testing, the stage micrometer was placed right above the prism to capture calibration images. During testing, the metal tube containing the prism was placed at the midpoint and underneath the muscle fibers. Starting from the initial position (i.e., no increase in the force on the tube due to muscle stretching), the tube was lifted up vertically step by step until the muscle fibers were stretched to 150% of the initial length.

A force microscope (Figure 1; Wu et al., 2016) that can estimate *in vivo* muscle tension (*via* its position and force sensors) and obtain sarcomere images was used to quantify the sarcomere length and tension of the soleus muscle in mice at varying times post-stroke. Plantar-flexors are commonly affected following stroke. When compared to the large gastrocnemius muscles in mice, the entire soleus muscle is small and thin enough to be easily isolated and placed on the top of the prism to view sarcomere images *in vivo* while minimizing muscle damage during dissection and/or muscle sample/bundle preparation.

**Figure 2:**
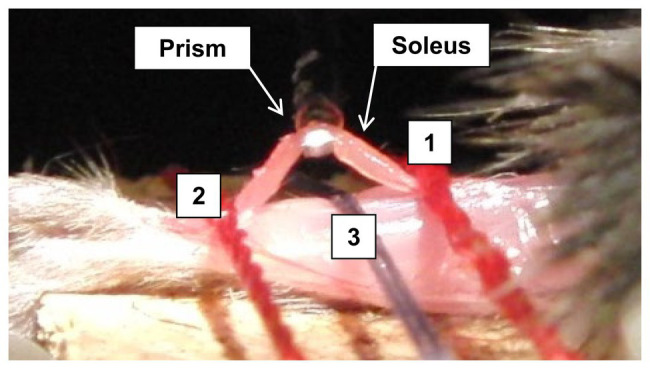
Illustration of the prism with light source underneath the isolated soleus bundle, where the isolated soleus muscle was held down during the biomechanical testing. Line 1 and line 2 are the position, where a suture holds down the isolated soleus muscle. Line 3 is the midpoint location, where another suture holds down the leg.

At the assigned time/day post-stroke, the mouse was anesthetized through intraperitoneal injection of xylazine (10 mg/kg) and ketamine (100 mg/kg). The mouse was laid on its stomach with the tested hindlimb secured/positioned at 0° of knee flexion and ankle dorsiflexion. The soleus muscle was then exposed and fixed at both ends close to the muscle-tendon junctions (Figure 2). Before testing, the initial muscle length, width, and thickness of the soleus muscle were measured.

Before testing, a stage micrometer (MA 285, Meiji Techno America, Santa Clara, CA, United States) was used to calibrate the pixel length of the subsequent captured images using this *in vivo* force microscope. The stage micrometer was placed right above the 0.5-mm prism contained in the tip of the metal tube, which also houses optical fiber transmitting the light from the light source and the objective was adjusted for focusing (Figure 1). A picture of the stage micrometer was then captured to be used for subsequent calibration when estimating the sarcomere length. Following image calibration, a 0.5-mm prism of the testing device was placed at the midpoint and under the isolated soleus muscle bundle. The initial vertical position of the prism was aligned with the soleus muscle to ensure no stretch in the muscle (i.e., determined based on whether an increase in force on the tube was detected) after the prism inserted in place. Images of the sarcomeres at the initial testing position were then obtained.

The sarcomere length of the soleus muscle at the initial testing position (i.e., 0° of knee flexion and 0° of ankle dorsiflexion) was measured from the obtained optic images. Specifically, ImageJ (National Institutes of Health, Bethesda, MD, United States; Schneider et al., 2012) was first used to measure the pixels of known distance of the gradation in the micrometer image. This calibration factor was then applied when the length of the sarcomere was measured from the recorded sarcomere images using ImageJ. At least 20 continuous sarcomeres were identified in each image to calculate the sarcomere length, and the average value from three images was used to represent the sarcomere length at the initial testing position. The entire muscle length was also measured. The sarcomere number in series was then estimated by dividing the muscle length by the average sarcomere length.

### *In vivo* Muscle Tension Estimation of the Soleus Muscle

Starting from the initial position, stepwise vertical lifting of the metal tube (i.e., right underneath the isolated soleus muscle) was conducted until the soleus muscle bundle was stretched to approximately 150% of the initial muscle bundle length (a total of 6–10 steps; approximately 0.3 mm/step; 1-min interval of each step). The vertical lifting force, vertical lifting distance, and images of the sarcomeres were simultaneously recorded *in vivo* at every lifting step. The recorded lifting force was used to estimate the axial tension of the muscle bundle at each lifting step. The lifting force was recorded continuously for the entire 1-min interval after each lifting step. However, the force signal fluctuated when a lifting step was first completed. Thus, to obtain a more stabilized force signal, only the forces recorded during the last 3-s window (i.e., 58th, 59th, and 60th s) were averaged and used for subsequent axial tension estimation at each lifting step.

**Figure 3:**
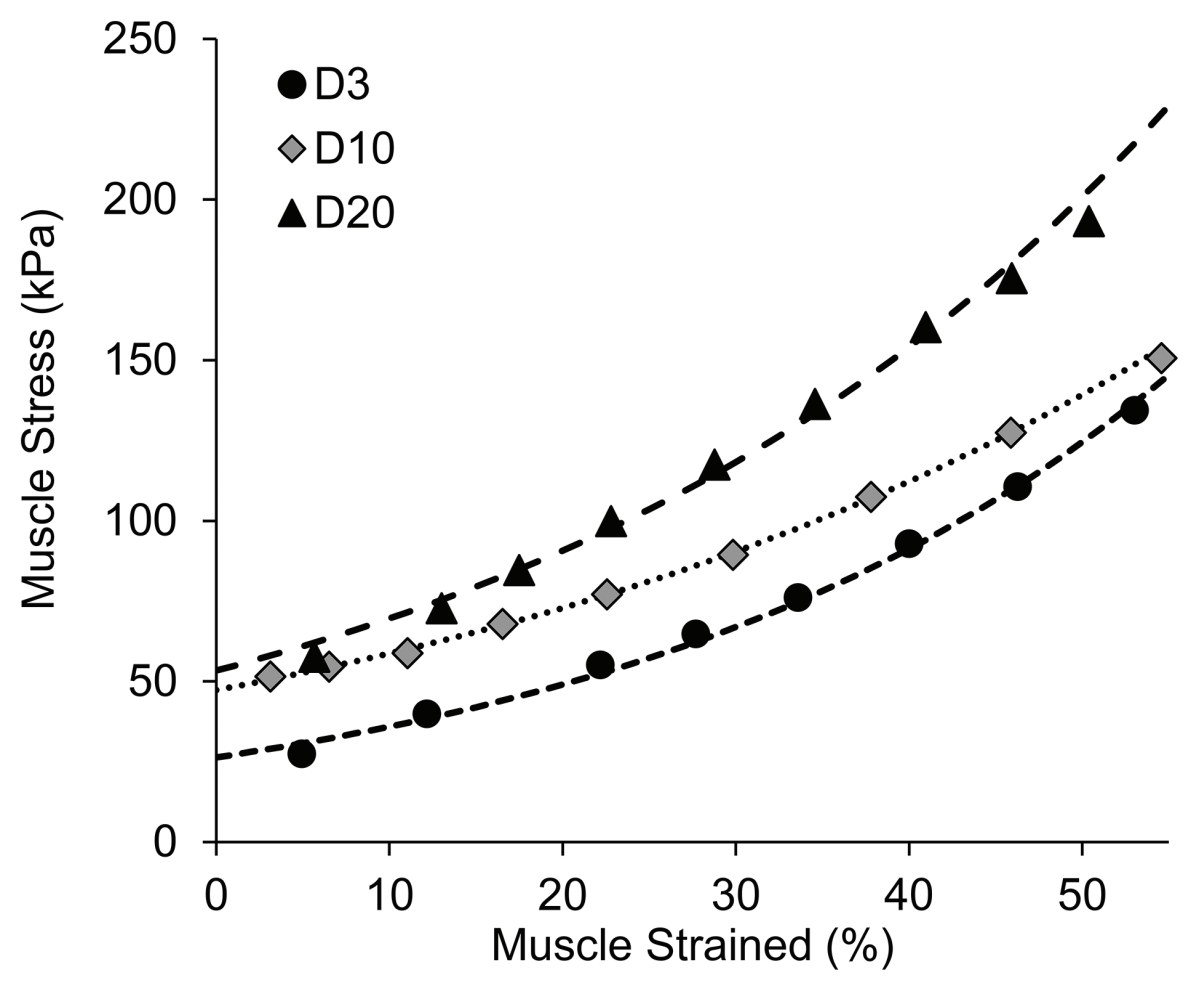
Representative samples showing the curve fitting procedures used to estimate the initial muscle stress.

The passive tension of the muscle at each lifting step was estimated using the recorded lifting force and vertical lifting distance based on trigonometry and force equilibrium calculations (Wu et al., 2016). Briefly, the passive muscle tension was estimated to be the lifting force divided by 2sin(*θ*), where *θ* is the angle formed by the horizontal line and the muscle bundle. Due to the limitation of this equation on estimating passive muscle tension at the initial length/step (i.e., when θ = 0, 1/sinθ = ∞), an exponential curve was fitted to the other data points to predict the passive tension of the muscle at the initial length (Figure 3). The predicted passive tension at the initial testing condition was normalized to the cross-sectional area of the muscle bundle to calculate the passive muscle stress (kPa).

### Data Analysis

The dependent variables included the initial sarcomere length, initial passive muscle stress (kPa), and the muscle density of the soleus muscle (g/cm^3^) in both the impaired limbs and less involved limbs. Two-way ANOVA (group-by-limb) with repeated measures was then used to examine the differences in each of the dependent variable among the three time-point groups and between the two legs with a significance level of *p* ≤ 0.05. If a significant interaction was detected, simple main effects analyses were conducted to examine each pair-wise comparison. If a significant group main effect was detected without a significant group-by-limb interaction, *post-hoc* between-group pairwise comparisons would be examined using Tukey’s HSD tests.

## Results

**Figure 4:**
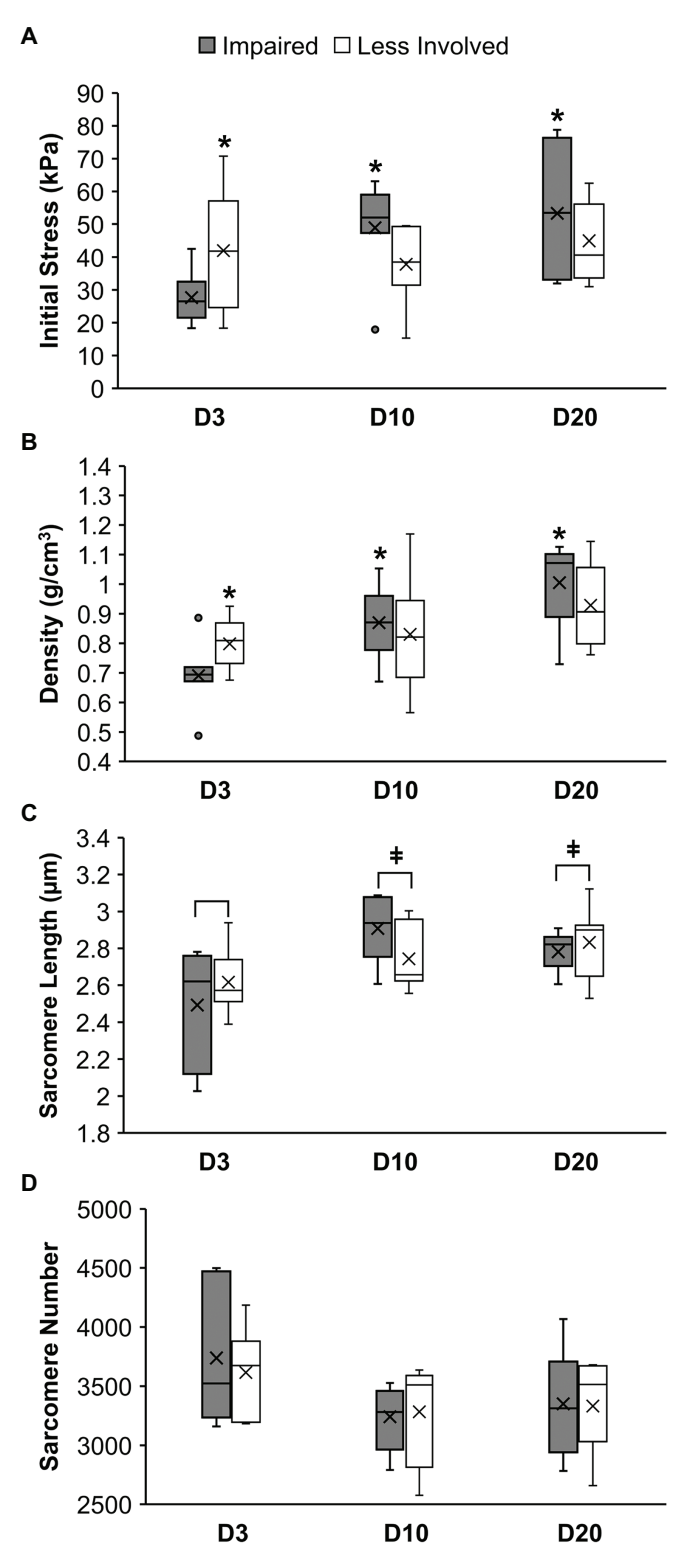
Muscle stress **(A)**, muscle density **(B)**, sarcomere length **(C)**, and sarcomere number **(D)** of the soleus muscle in mice at 3 days (D3), 10 days (D10), or 20 days (D20) following ischemic stroke. *Denotes a significant difference (*p* < 0.05) when compared to the impaired limb of the D3 group. ǂDenotes a significant difference (*p* < 0.05) when compared to the D3 group (i.e., both limbs combined).

Two-away ANOVA revealed a significant group-by-limb interaction for the initial passive muscle stress (*p* = 0.03; Figure 4A) and muscle density (*p* = 0.04; Figure 4B). *Post-hoc* simple main effects analyses indicated that the initial muscle stress of the impaired limbs in the D3 group (27.65 ± 8.37 kPa) was significantly smaller than muscle stress of the impaired limbs in the D10 group (48.92 ± 14.73; *p* = 0.03) and in the D20 group (53.28 ± 20.54 kPa; *p* = 0.01). No significant differences in the initial muscle stress among the three groups were observed in the less involved limbs (all *p* > 0.37). The muscle stress of the impaired limb was significant smaller than the muscle stress of the less involved limb in the D3 group (27.65 ± 8.37 vs. 42.03 ± 18.61 kPa; *p* = 0.05) but not in the D10 and D20 groups (*p* = 0.10 and 0.21, respectively).

Results of the *post-hoc* simple main effects analyses also indicated that the soleus muscle density of the impaired limbs in the D3 group (0.69 ± 0.12 g/cm^3^) was significantly smaller than the D10 (0.87 ± 0.12 g/cm^3^; *p* = 0.02) and D20 (1.00 ± 0.14 g/cm^3^; *p* < 0.01) groups. No significant differences in the muscle density among the three groups were observed for the less involved limbs (all *p* > 0.11). The muscle density of the impaired limb was significant smaller than the muscle density of the less involved limb in the D3 group (0.69 ± 0.12 vs. 0.80 ± 0.09 g/cm^3^; *p* = 0.04) but not in the D10 and D20 groups (*p* = 0.44 and 0.14, respectively).

For sarcomere length, two-way ANOVA only revealed a significant group main effect (*p* = 0.018; Figure 4C) without a significant group-by-limb interaction (*p* = 0.131). *Post-hoc* Tukey’s HSD tests indicated that the overall sarcomere length of the D3 group (2.55 ± 0.26 μm) was shorter than the sarcomere length of the D10 (2.83 ± 0.20 μm; *p* = 0.03) and D20 group (2.81 ± 0.15 μm; *p* = 0.04). While overall the D10 and 20 groups had less sarcomere number than the D3 group, results of the two-way ANOVA revealed no significant difference in the sarcomere number between limbs and among the three groups (*p* = 0.72 and 0.14, respectively; Figure 4D).

## Discussion

The overall objective of this study was to examine changes in sarcomere properties and muscle tension in mice following stroke. Using a force microscope, we were able to evaluate the *in vivo* morphology of the sarcomeres and muscle tension simultaneously at multiple time points following the brain surgery that induced ischemic stroke in mice. To our knowledge, limited information is currently available regarding the muscle fiber tension *in vivo* and the corresponding sarcomere morphology following neurological pathologies. The use of the established force microscope (Wu et al., 2016) thus demonstrates the potential to advance our understanding of the interaction between mechanical loads and morphological adaptation in skeletal muscles. It should be noted that the present study investigated the changes in muscles post-stroke under a passive condition, and thus future work that involves neural component (e.g., *via* electrical stimulation) as well as measures examining physiological and molecular mechanism is warranted to further understand the underlying mechanisms regarding the interactions between altered muscle tension and muscle morphological adaptation following neurological pathologies.

Alterations in the mechanical properties and sarcomere length of the skeletal muscle after a neurological disorder have been observed in children with CP (Smith et al., 2011; Dayanidhi and Lieber, 2014; Matthiasdottir et al., 2014; Mathewson and Lieber, 2015; Kinney et al., 2017). However, the time history of *in vivo* fiber tension and sarcomeric adaption after stroke has not been fully investigated. Our findings revealed marked changes in the passive muscle stress in mice following ischemic stroke, which are thought to trigger subsequent sarcomeric adaptation. Specifically, the observed passive muscle stress matched with the general pathology progression from the initial flaccid phase (i.e., decreased stress in D3) followed by increased tension due to the subsequent muscle hypertonia development (i.e., increased stress in D10 and D20) in the affected limb.

It has been proposed that sarcomerogenesis (sarcomere assembly) is achieved through ordered pathways (Ehler and Gautel, 2008) and may respond dynamically to mechanical stimulus to regulate the optimal operation range/length of the sarcomere (Herring et al., 1984). For example, animal studies have revealed that the sarcomere number increased in a lengthened position and decreased in a shortened position, and such adaptation is thought to be a response to the passive tension experienced by the muscle (Goldspink et al., 1974; Williams and Goldspink, 1978). Taken together, it was anticipated that the sarcomere number would start to decrease in response to a decrease in passive stress observed in D3 if sarcomeric adaptation occurred in mice following stroke. Thus, when the impaired soleus muscles were stretched to the initial testing position at 0° of ankle dorsiflexion, a longer sarcomere length as well as an elevated passive tension due to stretched sarcomeres would be expected. This premise was supported by the significant increase in the passive stress and sarcomere length in the D10 group when compared to the D3 group. While less sarcomeres were also observed in the D10 than the D3, this difference was not statistically significant.

However, no differences in passive stress, sarcomere length, and sarcomere number were observed between the D10 and D20 groups, suggesting that sarcomeric adaptation might be absent in mice at this stage of stroke. The elevated stress/tension of the soleus muscles when the ankle was held in a neutral position at D10 might lead to altered movement patterns and ankle joint positions (e.g., excessive plantar-flexion) adapted by the stroke mice in order to reduce the elevated muscle stress. This hypothesis may explain the absence of sarcomeric adaptation and thus the similarity between the D10 and D20 groups in all dependent variables. Future research that quantifies or controls the ankle joint kinematics/positions in mice following stroke is needed to determine whether sarcomeric adaptation in mice is disrupted during this stage of stroke. Regardless, the observed increased passive tension is expected to contribute to the limited joint motion and functions commonly observed following stroke.

A better understanding of the presence and mechanisms of sarcomeric adaptation is essential to assist in the decision-making and enhancing patient outcomes following clinical interventions in patients with neurological disorders. The impaired sarcomeric adaptation following CP has been associated with a reduction in the muscle satellite cells (Dayanidhi and Lieber, 2014; Mathewson and Lieber, 2015; Kinney et al., 2017). Future studies quantifying muscle satellite cells following stroke is warranted to examine whether the same underlying mechanism(s) are responsible for impaired sarcomeric adaptation across different neurological pathologies.

In addition to the changes in sarcomere length, the changes in the observed passive muscle stress in mice at different times post-stroke may also be associated with potential changes in muscle composition during the early stage of stroke in mice (Springer et al., 2014). This premise was supported by the differences in muscle density among the tested groups in our current study. The non-invasive ultrasonography method used in the patients with stroke also demonstrated the similar findings on the passive mechanical property changes after the neurological insults (Jakubowski et al., 2017; Leng et al., 2019). However, future research involving histological analyses of myofilament and cytoskeleton proteins will be needed to better understand how changes in muscle compositions post-stroke may potentially alter muscle stress/tension.

Several experimental limitations should be acknowledged. First, we estimated the volume of the muscle bundle using its length, width, and thickness based on the assumption of a cylinder. The estimation of the *in vivo* muscle axial tension/stress level using the force microscope also relied on the assumption of a constant initial length of the muscle bundle and achieving the force equilibrium status at every lifting step. The initial axial tension was predicted based on an exponential curve fitting procedure. Therefore, any violation of the above assumptions, such as poor fixation of the muscle and inaccurate curve fitting on the experimental data, would lead to measurement/prediction errors. However, we have carefully prepared the sample muscle with firm fixation on the two ends to prevent the potential errors. Muscle belly length was measured to calculate/estimate the sarcomere number of the soleus muscle. The muscle fiber length of rodents’ soleus muscles has been shown to be shorter than the muscle belly length (Burkholder et al., 1994; Tijs et al., 2015), and thus use of the muscle belly length, rather than the fiber length, would lead to an overestimation of the sarcomere number for all mice. The muscle testing involved the preparation of the soleus muscle bundle followed by multiple steps of vertical lifting might have also caused micro damages to the muscles, thereby potentially resulting in a lower stress value.

Additionally, the testing days of 3, 10, and 20 days post-stroke were selected simply based on our preliminary observations of stroke mice that demonstrated behavior changes representing the acute and subacute stages post-stroke (Liu et al., 2013). Thus, we might not be able to fully capture the changes in muscle tension and sarcomere morphology that occurred in other more critical time points post-stroke. We also did not include a control group to better minimize the potential impact of time on our data. The contralateral less involved limb can also be impacted by the stroke and thus may not serve as adequate controls (Heslinga and Huijing, 1992; Huijing and Maas, 2016). This may explain why we did not observe statistically significant differences between limbs beyond the acute stoke stage as in the D10 and D20 groups. Lastly, sarcomere plasticity is likely muscle dependent as revealed in a rat study, where different changes in sarcomere numbers were observed among the soleus and gastrocnemius muscles after the immobilization of the hind limb at various positions (Heslinga et al., 1995). This limits the generalizability of our findings to other impaired muscles.

## Conclusions

Changes in muscle passive tension and sarcomere length at multiple time points after ischemic stroke were examined *in vivo* using a mouse stroke model. Changes in muscle stress were accompanied with changes in sarcomere length at the early stage post-stroke. However, it remains unclear whether the process of sarcomeric adaptation in response to changes in mechanical stimuli may be compromised at the later stage of stroke. Future research with measurements of satellite cells, longer follow-up, and histological analyses of muscle compositions may help advance our understanding of the mechanisms and processes of muscle adaptation after ischemic stroke.

## Data Availability Statement

The raw data supporting the conclusions of this article will be made available by the authors, without undue reservation.

## Ethics Statement

The animal study was reviewed and approved by the Northwestern University Institutional Animal Care and Use Committee.

## Author Contributions

L-CT, Y-NW, and L-QZ contributed conception and design of the study. L-CT, Y-NW, and SL performed the experiments. L-CT wrote the first draft of the manuscript. All authors contributed to manuscript revision, read, and approved the submitted version.

### Conflict of Interest

The force microscope invention is covered in a joint patent between the Rehabilitation Institute of Chicago and Rehabtek LLC. L-QZ holds an equity position in Rehabtek.

The remaining authors declare that the research was conducted in the absence of any commercial or financial relationships that could be construed as a potential conflict of interest.
